# Characterization of muscle recruitment during gait of bilateral transfemoral and through-knee persons with limb loss

**DOI:** 10.3389/fbioe.2023.1128528

**Published:** 2023-04-04

**Authors:** Alice M. Benton, Pouya Amiri, David P. Henson, Biranavan Sivapuratharasu, Alison H. Mcgregor, Anthony M. J. Bull

**Affiliations:** ^1^ Department of Bioengineering, Imperial College London, London, United Kingdom; ^2^ Centre for Blast Injury Studies, Imperial College London, London, United Kingdom; ^3^ Department of Surgery and Cancer, Imperial College London, London, United Kingdom

**Keywords:** transfemoral bilateral limb loss, muscle endurance, muscoskeletal modelling, gait, biomechanics

## Abstract

**Introduction:** Due to loss in musculoskeletal capacity, there is an increased burden on the residual limbs of bilateral transfemoral and through-knee persons with limb loss. This reduced capacity is associated with an increased cost of walking that is detrimental to functionality. Compensatory gait strategies are adopted by this population. However, how these strategies relate to specific muscle recruitment is not known. The primary aim of this study is to characterize muscle recruitment during gait of this population. The secondary aim is to assess whether the measured kinematics can be actuated when the endurance of specific muscles is reduced and if this is the case, which alternative muscles facilitate this.

**Methods:** 3D gait data and high-resolution magnetic resonance images were acquired from six bilateral transfemoral and through-knee persons with limb loss. Subject-specific anatomical muscle models were developed for each participant, and a validated musculoskeletal model was used to quantify muscle forces in two conditions: during normal gait (baseline) and when muscles, which were identified as functioning above a “healthy” level at baseline, have a reduced magnitude of maximum force capacity (reduced endurance simulation). To test the hypothesis that there are differences in muscle forces between the baseline trials and the simulations with reduced muscular endurance, a Bonferroni corrected two-way ANOVA with repeated measures was completed between the two states.

**Results:** The baseline analysis showed that the hip flexors experience relatively high muscle activations during gait. The reduced endurance simulation found two scenarios. First, for 5 out of the 12 simulations, the baseline kinematics could not be reproduced with the reduced muscular capacity. Second, for 7 out of 12 cases where the baseline kinematics were achieved, this was possible with compensatory increased activation of some muscles with similar functions (*p* ≤ 0.003).

**Discussion:** Evidently, due to the loss of the ankle plantar flexors, gait imposes a high demand on the flexor muscle group of the residual limb. This study highlights how the elevated cost of gait in this population manifests in muscle recruitment. To enhance functionality, it is critical to consider the mechanical demand on the hip flexors and to develop rehabilitation interventions accordingly.

## 1 Introduction

The partial loss of a lower limb manifests as a loss in neuromusculoskeletal capacity. The central nervous system will adapt to this loss through the adoption of compensatory strategies which enable a person with limb loss (PWLL) to ambulate (Hoffman et al., 1997; Su et al., 2007; Visser et al., 2011; Visser et al., 2011; van der Kruk et al., 2021). High levels of functionality for lower-limb PWLLs are possible and have been observed in the ex-military population when comprehensive rehabilitation interventions were completed and state-of-the-art prosthetics used (Ladlow et al., 2015; Jarvis et al., 2017; Jarvis et al., 2021). However, gait remains more functionally demanding for bilateral transfemoral PWLLs compared to able-bodied persons (Hoffman et al., 1997), with lower scores found in 6 min walking tests (Linberg et al., 2013) and higher metabolic cost during gait (Jarvis et al., 2017) in the young ex-military population.

Compensatory gait strategies, emerging as a result of amputation, result in high muscle forces that lead to elevated hip joint contact forces (Su et al., 2007; Wentink et al., 2013). Increased muscle activations place a large physiological demand on the muscle of the residual limb, likely increasing energy expenditure and having a detrimental effect on endurance (Fang et al., 2007), rendering PWLLs less able to perform recreational activities and activities of daily life (Wong et al., 2015). However, how the compensatory gait strategies relate to specific muscle recruitment patterns is not known.

Muscular endurance is defined as the ability of a muscle to execute extended repetitions of muscle contractions (Kell et al., 2001). With repeated muscle contractions, as occurs during walking, there will be a reduction in muscle performance associated with a reduction in the muscle’s ability to produce force (Van Geel et al., 2021). Experiments on isolated muscle cells demonstrate that contractions near maximum capacity induce a reduction in the magnitude of the force of the contractions (Bishop, 2012). The reduction in maximum voluntary contraction (MVC) of intact muscles during tasks has been explained by metabolic accumulation and impaired motor control (Bishop, 2012). Muscular endurance of lower-limb muscle during gait has been quantified for healthy subjects by an observed reduction in the medium frequency of EMG signals (Fidalgo-Herrera et al., 2022). An experiment that implemented “fatigue protocols” on lower-limb muscles, consisting of a repeated sit-to-stand task, reported that muscle endurance affects spatiotemporal and kinematic features of gait in healthy subjects (Barbieri et al., 2013). These changes in gait characteristics increase the risk of falling (Mohd Safee and Abu Osman, 2021) and localized tissue injury (Kumar, 2001). Endurance is dependent on the intensity of muscle contractions (West et al., 1995). Overall, high-intensity muscle forces will limit a PWLL’s functionality as they will be detrimental to lower-limb muscular endurance during gait.

Musculoskeletal (MSK) modeling enables muscle and articular contact forces to be estimated. Inverse MSK modeling uses the observed kinematics and ground reaction forces to determine the net forces and moments at the joints and uses an optimization method to resolve the muscle force sharing. FreeBody is a segment-based MSK model (Cleather and Bull, 2015) that has been extensively validated for muscle activations using EMG experimental data for bilateral transfemoral amputees (Toderita et al., 2021), unilateral transtibial amputees (Ding et al., 2023), knee joint contact forces for osteoarthritis patients (Ding et al., 2016), and hip joint contact forces also using instrumented implants (Amiri and Bull, 2022).

Able-bodied walking is an optimized activity that minimizes muscle recruitment (Crowninshield and Brand, 1981). Therefore, this can provide a gold standard for the development of muscle coordination strategies for improved endurance. Muscle activation can be estimated using experimental EMG data and normalized to EMG at MVC. Healthy walking recruits lower-limb muscles generally to below or up to 50% of their MVC (Burden et al., 2003; Nishijima et al., 2010; Franz and Kram, 2013; Guan et al., 2019; Elsais et al., 2020), with many values in the literature being considerably lower than 50%. A range between 5%–64% has been reported for different muscles with an average value of ∼ 27%. The demand of gait on individuals has also been quantified by net torque actuated across joints as a percentage of maximal torque capacity. Two studies have quantified maximal torque produced in hip flexion and hip extension for self-selected walking. One study quantified this value to be below 30% in flexion and extension in young adults (*n* = 10) (Anderson and Madigan, 2014). The other found the percentage of maximum torque capacity produced during gait to be below 50% in the hip flexors and below 35% in the hip extensors for adults (*n* = 14) (Requiao et al., 2005). Both studies found that this metric of “functional capacity utilized” increased with increased cadence.

The first objective of this study was to quantify the muscle forces in bilateral TFTK PWLL gait. The second objective was to determine the impact of reduced endurance, simulated by reducing specific muscle force capacities, on this group’s ability to actuate their baseline kinematics and to identify potential compensatory muscle strategies. Both objectives aim to increase the understanding of muscle recruitment strategies in this population to inform future rehabilitation research.

## 2 Materials and methods

### 2.1 Participants

This study focused on the young ex-military population as a model of the highest potential functional outcome (Jarvis et al., 2021). They are young, have had comprehensive rehabilitation, and are provided with state-of-the-art prosthetics. As lower-limb anatomical and biomechanical factors are different between sexes, a mixed group should include equal numbers. This was not possible as this population has few female PWLLs. Therefore, this study looked at only male participants. A total of 6 male (aged 27–36 years) bilateral TFTK PWLLs due to trauma were included in this study. All subjects were fitted with their own state-of-the-art mechanical prosthetic feet and microprocessor prosthetic knees. Subject details are presented in Table 1. Ethical approval was received from the institutional ethics review board, and written informed consent was obtained from the participants.

**TABLE 1:** Bilateral persons with limb loss participant details, TK = through-knee and TF = transfemoral.

Subject	Sex	Body mass (kg)	Age (years)	Height (cm)	Left-side level and code	Right-side level and code	Prosthetic foot	Prosthetic knee
**1**	Male	86.0	32	185	TK	TF	Triton low profile	Genium
					1L	1R		
**2**	Male	81.2	27	178	TK	TF	Triton	Genium X3
					2L	2R		
**3**	Male	90.0	36	186	TK	TF	Triton low profile	Genium X3
					3L	3R		
**4**	Male	89.5	33	182	TK	TK	Triton low profile	Genium
					4L	4R		
**5**	Male	97.2	32	185	TK	TK	Triton	Genium X3
					5L	5R		
**6**	Male	68.8	31	171	TF	TF	Triton	Genium X3
					6L	6R		

### 2.2 Gait data collection

Three self-selected gait trials and one static calibration trial were collected per participant in a gait analysis laboratory consisting of a motion capture system with 10 cameras (VICON, Oxford Metrics Group, United Kingdom) and two force plates (Kistler Type 9286B, Kistler Instrumented AG, Winterthur, Switzerland). The marker 3D displacement and ground reaction forces were sampled at 120 and 960 Hz, respectively. Reflective markers were attached to 14 anatomical landmarks, as listed in Table 2, on both the left and right legs, as well as two clusters of markers on the thigh and calf of both limbs (Toderita et al., 2021).

**TABLE 2:** Optical motion tracking cluster and marker positions and labels.

Marker label	Anatomical location
FCC	Calcaneus
FMT	Tuberosity of the fifth metatarsal
FM2	Head of the second metatarsal
TF	Additional marker placed on foot
FAM	Apex of the lateral malleolus
TAM	Apex of the medial malleolus
C1,C2, C3 (clusters)	Additional markers placed on the shank segment
FLE	Lateral femoral epicondyle
FME	Medial femoral epicondyle
T1,T2, and T3 (clusters)	Additional markers placed on the thigh segment
RASIS	Right anterior superior iliac spine
LASIS	Left anterior superior iliac spine
RPSIS	Right posterior superior iliac spine
LPSIS	Left posterior superior iliac spine

### 2.3 Anatomical dataset

Anatomical datasets of all participants have previously been published (Toderita et al., 2021). In summary, these datasets were built from MRI scans acquired with a 3.0T MRI scanner (MAGNETOM Verio, Siemens, Germany) with a 3D T1-weighted spoiled gradient echo sequence set at a 450 × 450 ${mm}^{2}$ field of view, 1 mm slice thickness, and 1.17 × 1.17 mm axial plane resolution. The scans were completed from the iliac crest to the distal end of the longest residuum, so that an anatomical atlas could be constructed of the left and the right residual limb of each of the 6 participants (Toderita et al., 2021).

### 2.4 Subject-specific MSK modeling

The FreeBody model (Cleather and Bull, 2015) was utilized to quantify the muscle forces during the gait trials. FreeBody consists of four segments: the foot, shank, thigh, and pelvis, which are connected through the ankle, hip, and knee joints. The residuum, socket, and liner are modeled as the thigh segment. The transfemoral PWLL MSK model contains 92 muscle elements, representing 21 muscles. The subject-specific geometry of muscle and bones was obtained from the MRI images, the details of which are outlined elsewhere (Toderita et al., 2021) and are described here briefly. For each leg, the MRI scans of the subject were used to render 3D shapes of bones and muscles. Subsequently, following the methodology of Horsman et al., 2007, the muscle origins, insertion and *via* points, hip joint rotation center, and a cylindrical wrapping surface (to represent iliopsoas muscle elements actions along superior pubic ramus of the pelvis) were digitized.

The physiological cross-sectional area (PSCA) of each muscle was calculated using the following equation[Disp-formula e1]:

$$PCSA=\frac{V_{m}\times \cos\theta }{L_{m}\times \frac{L_{f}}{L_{m}}\times \frac{2.7}{L_{s}}},$$ (1)

where muscle volume ( $\left.V_{m}\right)$ was acquired from rendered 3D geometry of the muscles, muscle length to its length ratio ( $\frac{L_{f}}{L_{m}}$ ), pennation angle ( θ ), and optimal sarcomere length ( $L_{s}$ ) were taken from the literature. In the cases where there were no values found in the literature, θ was set to 0, $\frac{L_{f}}{L_{m}}$ was set to 1, and $L_{s}$ was set to 2.7 μ m (Toderita et al., 2021).

The maximum force potential ( $F_{\max}^{i}$ ) of each muscle was calculated from the multiplication of its PCSA (which is subject specific, as per the anatomical atlas) and an assumed fixed maximum muscle stress (31.39 N $/{cm}^{2}$ ), following the equation (Yamaguchi, 2005)

$$F_{\max}^{i}={PCSA}_{i}\;\times \;M_{i}\;\times \sigma ,$$ (2)

where $F_{\max}^{i}$ , $M_{i}$ , and σ represent the maximum force potential, multiplier, and maximum muscle stress, respectively. The multiplier ( $M_{i}$ ) was used to modify $F_{\max}^{i}$ to account for changes in muscle structure that are known to occur due to atrophy and hypertrophy in the residual limb of a transfemoral PWLL post-surgery and rehabilitation (Henson et al., 2021; Toderita et al., 2021). There are two contributing factors to the progression of atrophy and hypertrophy observed. First, changes in muscle recruitment in the monoarticular function are assumed by muscles that have a biarticular function of the knee and hip in their intact state (Henson et al., 2021). Second, the direct effects of the surgery can produce atrophy in muscles that have been re-attached to a non-physiological site (Fraisse et al., 2008; Putz et al., 2017). Therefore, $M_{i}$ of the muscles which are distally inserted from the site of surgery was set to less than 1, with muscles more affected by the surgery set lowest. As per these observations, $M_{i}$ was reduced to the following: 0.12 for rectus femoris, 0.33 for bicep femoris and gracilis, 0.50 for the adductor magnus and semimembranosus, and 0.80 for the tensor fascia latae and sartorius. As the adductor brevis, iliacus, pectineus, and gluteus medius have a greater function in the compensatory gait of a PWLL (Henson et al., 2021), $M_{i}$ of these muscles was set to 1.20. The adaptations of $F_{\max}^{i}$ account for the physiological/structural changes that occur in an atrophied/hypertrophied muscle which affect force capacity but are not represented by the changes in muscle volume (Henson et al., 2021).

Quaternion algebra was used to perform inverse kinematic calculations, and inverse dynamics simulations were carried out using a wrench formulation to estimate the net forces and moments during the captured motion (Dumas et al., 2004). The model then ran a one-step constraint static optimization to estimate muscle and articular contact forces over the captured gait cycle. The load-sharing optimization minimized the sum of the cubed muscle activations, as shown in Eq 3, while satisfying the equations of motion of the MSK system and constraining the muscle forces to their maximum force potentials:

$$J=\sum_{i=1}^{92}{\left(\frac{F_{i}}{F_{\max}^{i}}\right)}^{3},$$ (3)

where J and $F_{i}$ are the sum of cubed muscle activations and force of muscle element I, respectively.

Two sets of simulations were performed: first, to identify overburdened muscles during gait (*baseline*) and then to quantify the consequences of the reduced muscular endurance of the overburdened muscles (*reduced endurance*).

#### 2.4.1 Baseline PWLL gait simulations

Captured gait kinematics and kinetics, along with the subject-specific MSK geometry, were used to estimate muscle forces. Muscles whose mean activations ( $F_{i}$ / $F_{\max}^{i}$ ) across the three trials for all legs were above the maximal level in healthy gait were identified as overburdened muscles. The threshold for healthy muscle activation during gait was taken as 50% force capacity (0.5 $F_{\max}^{i}$ ), as reported in previous EMG (Burden et al., 2003; Nishijima et al., 2010; Franz and Kram, 2013; Guan et al., 2019; Elsais et al., 2020) and maximal torque studies (Requiao et al., 2005; Anderson and Madigan, 2014). The term “overburdened” will be used throughout this study to refer to muscles that have activations higher than this threshold.

#### 2.4.2 Reduced muscular endurance PWLL gait simulations

Muscular endurance is defined by the ability of a muscle to maintain a level of muscle contraction over extended repetitions (Kell et al., 2001). A reduced muscular endurance can, therefore, be quantified by a reduced force production capacity during a task of repeated contractions (i.e., gait). A reduced muscular endurance was, therefore, simulated by re-running the optimizations with reduced maximum force potentials ( $F_{\max}^{i})$ of the muscles which were identified as overburdened from the results of the baseline gait simulations. The $F_{\max}^{i}$ of these muscles were reduced to 50% of their original level. It is hypothesized that inhibitory senses prevent a level of critical reduced muscular performance from occurring, meaning that task failure (i.e., the inability to maintain baseline kinematics in this case) will occur before a subject-specific critical level of reduction in MVC is reached (Amann and Dempsey, 2008). In the practice of modeling reduced muscular endurance, this suggests that modeling a reduction of MVC to a point beyond 50% may risk being physiologically irrelevant due to a protective mechanism of the body.

The *reduced endurance* simulations were carried out on one gait trial per subject; the trial selected was the trial with the highest maximum force of the ‘overburdened’ muscles from the baseline gait optimization. It was investigated whether the optimization was able to find a feasible solution (i.e., whether the reduced muscle capacity can reproduce the experimentally collected baseline kinematics). If a feasible solution could be found, it would likely be due to a compensatory muscle recruitment strategy (van der Kruk et al., 2021). It was hypothesized that muscles with a similar function would indicate a significantly greater muscle recruitment force in the reduced endurance condition compared to baseline.

Normal distribution of the maximum muscle activations (force/ $F_{\max}^{i})$ for each muscle was assumed, and a two-way repeated measures ANOVA was performed with SPSS software between the maximum muscle activations of *reduced endurance* simulations (for the legs where a solution could be found in the *reduced endurance* simulations) and *baseline* (only including the baseline trials used in the *reduced endurance* simulations, for the legs where a solution could be found). The nominal variables were muscle type and condition (*baseline* and *reduced endurance*), the test included 13 main muscles (excluding the muscles with simulated reduced force capacities), and alpha was adjusted with Bonferroni correction for pairwise comparisons. Each leg was treated as independent in the statistical analysis due to the variable trauma-induced anatomy.

## 3 Results

### 3.1 Example plot

**FIGURE 1:**
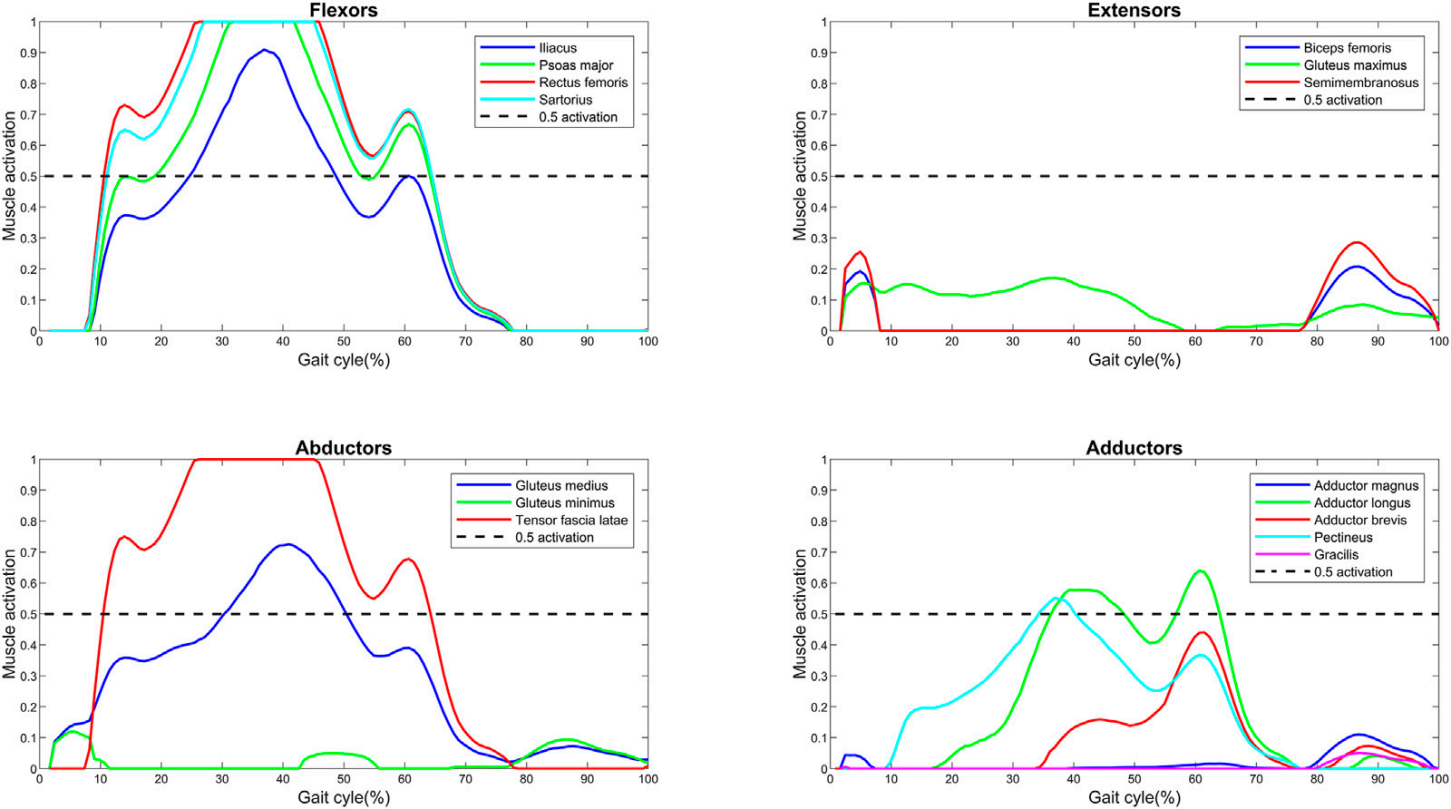
Muscle activations (force normalized to $F_{\max}^{i})$ over a gait cycle for an individual with bilateral transfemoral limb loss. Muscles are grouped by primary function: flexors, extensors, abductors, and adductors.

The *baseline* muscle activations (F/ $F_{\max}^{i}$ ) are plotted over the gait cycle (1%–100%). An example plot for a trial of a subject is displayed in Figure 1. The baseline gait trial was plotted for the left leg of participant 2 (2L), as this was a typical example of muscle activation. The main muscles are shown grouped by primary function (flexors, extensors, abductors, and adductors). In this example, it can be observed that the psoas major, rectus femoris, sartorius, and tensor fascia latae become saturated (activation reaches 1.0, meaning that they have reached the limit of their theoretical load-generating capacity, as defined by the physiological cross-sectional area and maximum muscle stress). Additionally, the iliacus, gluteus medius, and adductor longus muscles are functioning at greater than 0.5 activations.

### 3.2 Characterizing the muscle overloading

**FIGURE 2:**
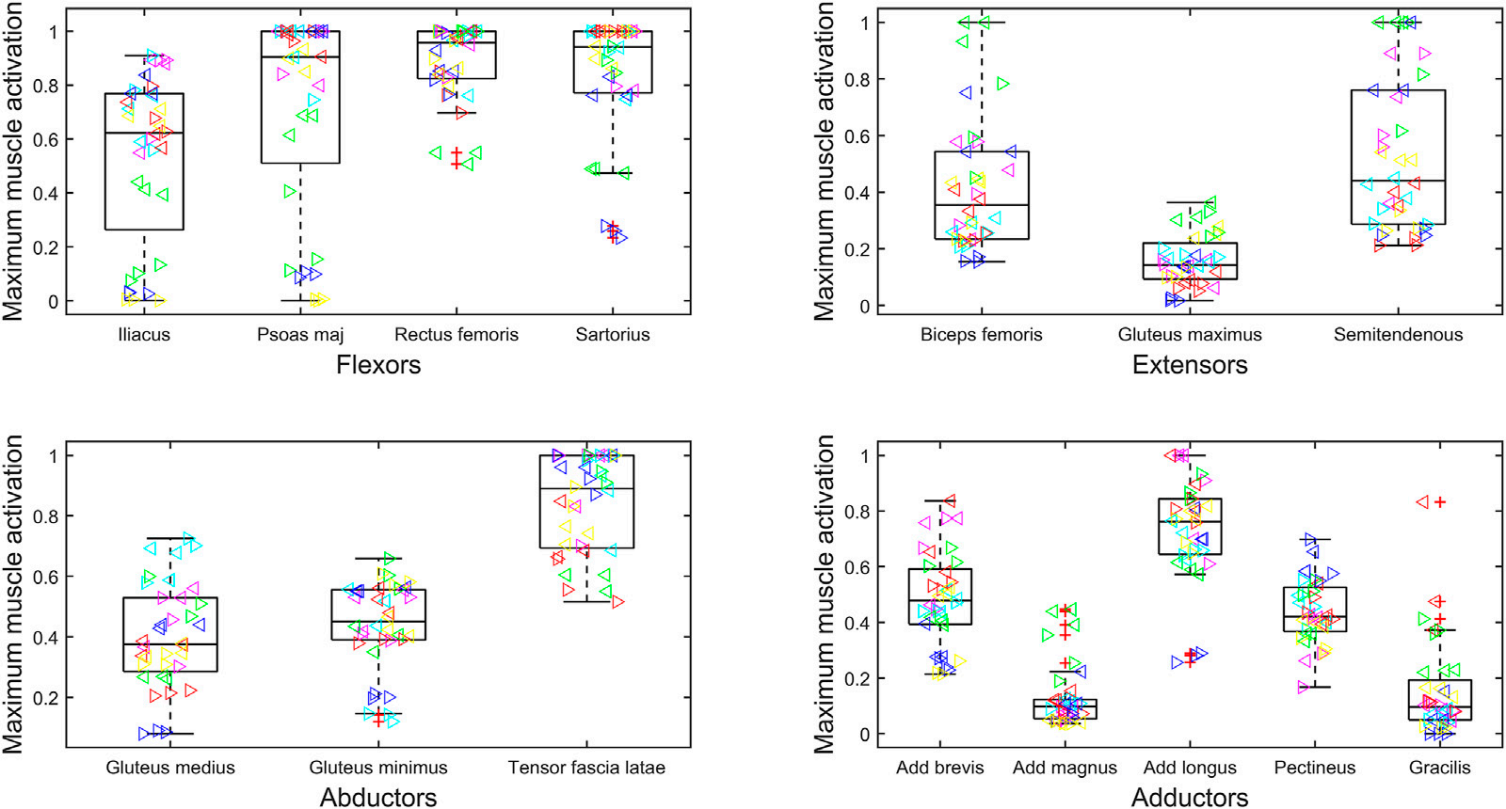
Maximum muscle activation (force normalized to $F_{\max}^{i})$ during gait cycle of bilateral transfemoral persons with limb loss. Muscles are categorized as hip flexors, extensors, abductors, and adductors. The box plots show min, max, median, 25 and 75 percentiles, and the outliers. Each subject is plotted in a separate color, and the left legs are represented by ‘►’ and right by ‘◄’.

**FIGURE 3:**
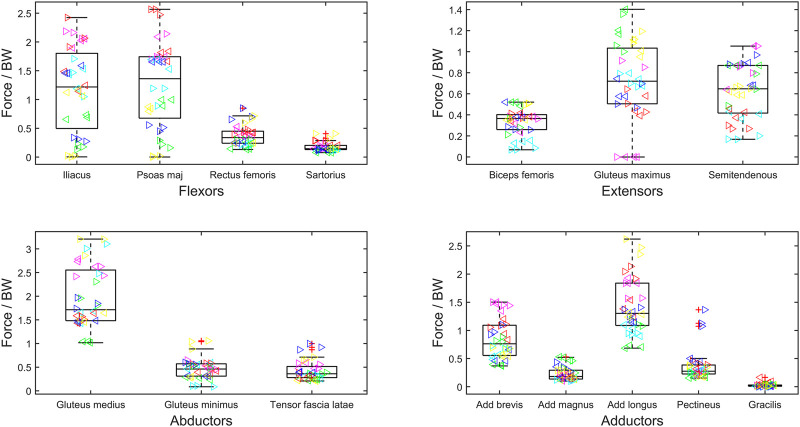
Maximum muscle force normalized to body weight (BW) during gait cycle of bilateral transfemoral persons with limb loss. Muscles are categorized as hip flexors, extensors, abductors, and adductors. The box plots show min, max, median, 25 and 75 percentiles, and the outliers. Each subject is plotted in a separate color, and the left legs are represented by ‘►’ and right by ‘◄’.

The maximum force generated by the muscles of the residual limb has been normalized to $F_{\max}^{i}$ (i.e., muscle activation) in Figure 2 and subject-specific body weight (BW) in Figure 3. The muscles are grouped by primary functions. The median activation of the flexor muscle group is high (above 0.5), and this is also true for the tensor fascia latae and adductor longus (Figure 2). For muscles that have an average maximum muscle activation above 0.5 (psoas major = 0.7, adductor longus = 0.7, rectus femoris = 0.9, sartorius = 0.8, and tensor fascia latae = 0.9), the adductor longus and psoas major also have larger normalized magnitudes (Figure 3), suggesting that their force contributions are instrumental in creating the moments required by gait.

### 3.3 Reduced endurance simulation

Following the *baseline* characterization, the psoas major and the adductor longus were identified as muscles which would be particularly susceptible to experiencing reduced endurance (as they have high activations and high magnitudes); their $F_{\max}^{i}$ was reduced to 50%. In the *reduced endurance* condition, it can be predicated that three out of the six participants could not retain their *baseline* kinematics, as the optimization could not find a solution for five out of the twelve legs studied. This was true for both the sides of subjects 1 and 5 and the right limb of subject 6. For the following legs, a solution could be found in the reduced endurance state: 2L, 2R, 3L, 3R, 4L, 4R, and 6R. In the legs where a solution could be found (7/12), there was a consistent increase in total maximum muscle activation of the muscles over the gait cycle, with the increase ranging from 3.3% to 30.4% in total maximum activation in the *reduced endurance* condition (the average increase was 19.7%). The total maximum muscle activation is the sum of the maximum muscle activation reached over the gait cycles by all 21 muscles modeled.

**FIGURE 4:**
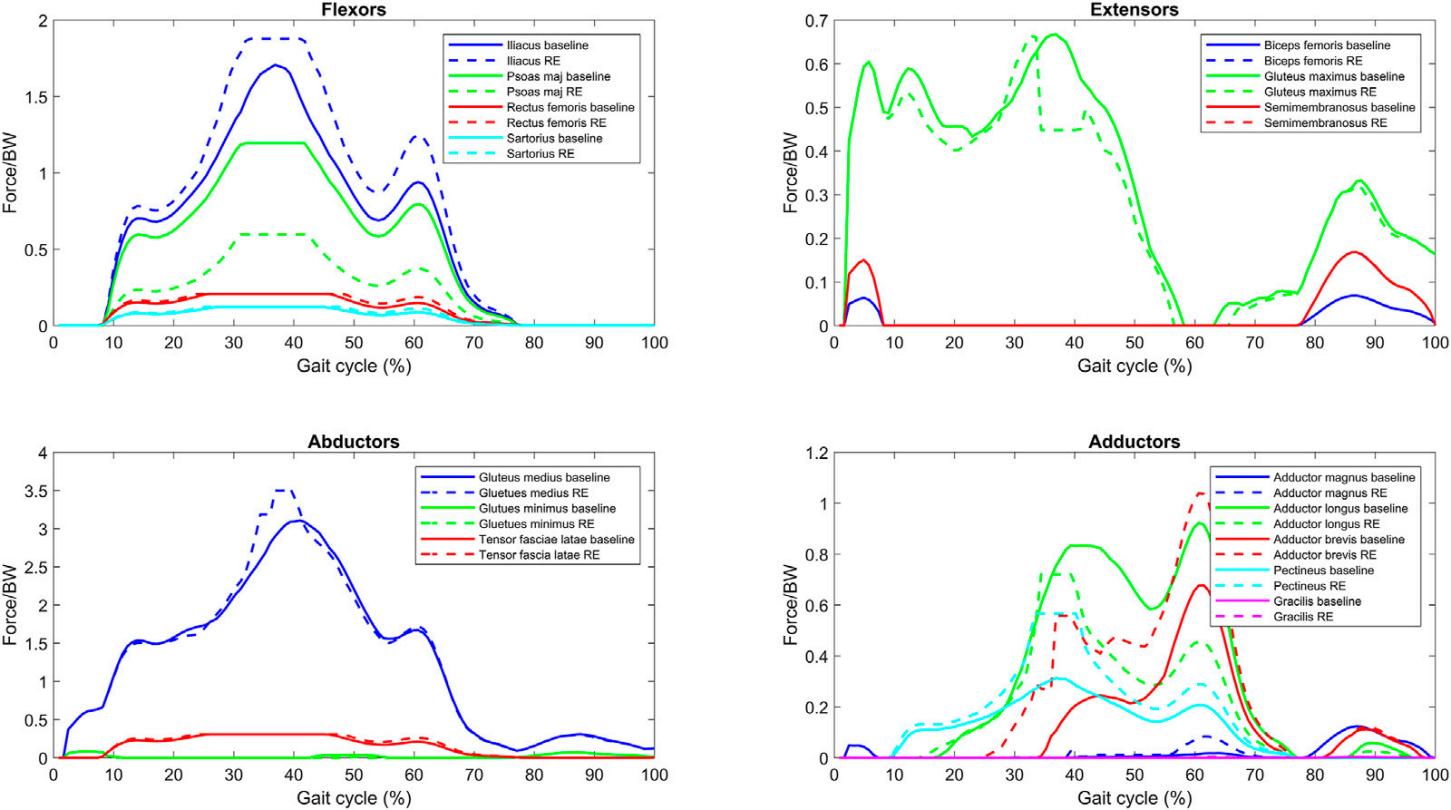
Normalized muscle force for one bilateral transfemoral person with limb loss for baseline and reduced endurance (RE) condition gait trial (reduce force capacity of the psoas major and adductor longus).

An example plot of the *reduced endurance* condition is presented in Figure 4. The leg plotted in this example is the left leg of subject 2 (2L). This example shows that the simulated reduced force capacity of the psoas major and the adductor longus increases the force contributions of the remaining muscles of the same primary function.

**FIGURE 5:**
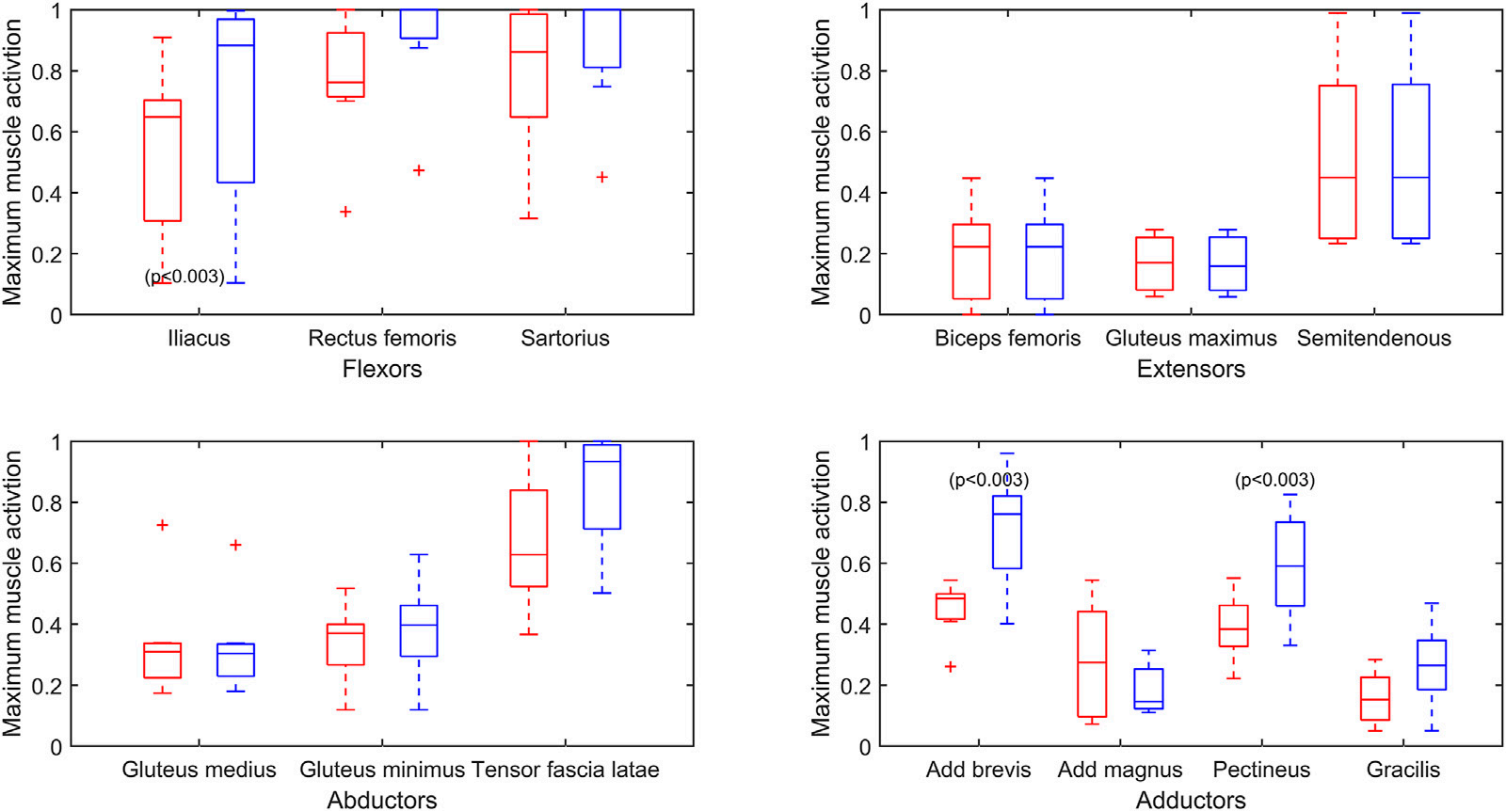
Maximum muscle activation (force/ $F_{\max}^{i})$ over a gait cycle for bilateral transfemoral persons with limb loss participants (baseline in red, reduced endurance state in blue). Baseline values are plotted for only legs for which a feasible solution could be found in the reduced endurance state. A reduced endurance state simulated by a reduction in the maximum capacity of the psoas major and the adductor longus is shown. Muscles are grouped into primary function; *p* values are presented on a plot for significant difference between the means of baseline and the reduced endurance condition.

Figure 5 presents the overall effect of the *reduced endurance* condition on the maximum activations (force normalized to $F_{\max}^{i}$ ) of the remaining muscles when the psoas major and the adductor longus have reduced force capacities. The muscles are grouped by primary function. All legs where a full solution could be found in the *reduced endurance* optimizations were included in this plot for *baseline* and *reduced endurance* (2L, 2R, 3L, 3R, 4L, 4R, and 6R). All legs where a full solution could not be found were excluded (1L, 1R, 5L, 5R, and 6L). The maximum activations (force normalized to $F_{\max}^{i}$ ) are plotted for the *baseline* (red) and the *reduced endurance* (blue) simulations. In the simulations of reduced muscular endurance of the psoas major and adductor longus, the maximum activations (force normalized to $F_{\max}^{i}$ ) of the iliacus, adductor brevis, and pectineus are increased (*p* ≤ 0.003). This is indicative of the increased burden on these muscles when the psoas major and the adductor longus have reduced endurance.

## 4 Discussion

In agreement with the literature, this study has shown that bilateral TFTK PWLL gait results in highly burdened flexor muscles due to the loss of ankle propulsion (Mcnealy And Gard, 2008). Although not their primary function, the adductor longus and the tensor fascia latae provide some flexor action (Neumann, 2010), and these also have high activations in this study. This study has highlighted the high cost of gait on the residual MSK system of this young traumatic TFTK PWLL population, highlighting the importance of the preservation of all residual flexor action capacity in surgery and flexor functionality in rehabilitation, as well as its consideration with aging. This high cost is particularly significant for the psoas major and adductor longus, as their critical role in function is demonstrated in the *reduced endurance* simulation in this study, with 5 out of the 12 simulations not being able to find a feasible solution and preserve baseline kinematics with the reduced capacity of these two muscles. Their function should, therefore, be given emphasis in rehabilitation protocols.

Our estimations of muscle force found the average activation of the flexor muscles to be above 50% of their maximum force potential. Lower-limb PWLLs experience a loss in muscular capacity. Therefore, daily tasks are executed close to their functional capacity (van der Kruk et al., 2021). The effect of this specific loss in capacity on muscular endurance seems unexplored in published studies in the context of lower-limb PWLLs. However, this is comparable to the loss in muscular capacity of the elderly population, which has been much more comprehensively studied. In studies of the elderly population, muscle activation (EMG/MVC) has been taken as a measure of work relative to capacity. It is concluded that the elderly population has greater difficulty with performing daily activities due to experiencing early-onset fatigue because the activities require performance that is closer to their functional capacity than the younger population (Hortobágyi et al., 2003; Hahn et al., 2005; Hortobágyi et al., 2011). For example, through an EMG experimental set up, one study found that level walking required 80.2% MVC in the elderly group compared to 37.5% MVC in the younger group when quantifying the sum of muscle activation for the vastus lateralis, biceps femoris, tibialis anterior, and the lateral gastrocnemius (Hortobágyi et al., 2011). Another study by the same author highlights how this difference in muscle activity with respect to muscle capacity between the old and the young is true for other activities of daily life for example. Similar differences were found in ascending (62% and 28% MVC) and descending (79% and 33% MVC) stairs between the elderly and younger participants, respectively, when just studying the vastus lateralis (Hortobágyi et al., 2003). We have used a similar method of analyzing the comparative relative demands of walking, and these studies consolidate the need for a rehabilitation method to relieve the flexor muscles with respect to their capacity, to reduce the risk of detriment to muscular endurance and reflect how our findings may be applicable to other activities of daily life.

High inter-subject variability within muscle forces and high inter-subject variance in kinematics and, therefore, resultant loading has been reported previously in the bilateral population (Wright et al., 2008; Wentink et al., 2013). In our study, this is particularly evident when looking at the maximum muscle activations of the psoas major and iliacus (Figure 2). For some participants, the muscles appear to become saturated, and in other participants, they are only minimally recruited. This variation suggests the need for patient-specific prescription of interventions and highlights potential underlying differences in gait biomechanics.

Our baseline gait simulations found that the psoas major and the adductor longus function at comparatively high activations and magnitude. High-intensity muscle contractions will induce a reduced muscular performance (Kumar, 2001; Westerblad and Allen, 2002), so when selecting muscles that may experience reduced endurance, we chose the muscles with both high activations and high absolute forces as these will have a larger contribution to the moments of gait. Muscles that function at high activation but low relative absolute magnitude may experience a decline in performance, but this will be less detrimental to gait. Our simulations of reduced endurance of the psoas major and adductor longus demonstrated two scenarios. The first, which was true for half the subjects, is that the original kinematics were not possible with the reduced contribution of psoas major and adductor longus. This corroborates our findings that these muscles are essential for maintaining gait of bilateral TFTK PWLLs, and in these cases, a kinematic adaptation is required. The second is where a full solution could be found despite the simulated reduced capacity of the adductor longus and psoas major. This occurred in 7 out of 12 simulations, highlighting redundancy in the MSK system and therefore robustness to a reduced endurance of the overburdened muscles, as shown previously (Komura et al., 2004). This was achieved by compensating for the impaired muscles by the recruitment of muscles with similar functions. Muscle recruitment in the *reduced endurance* state requires high forces of the compensating muscles, which in turn increases the acute functional demand on these muscles. This study included both transfemoral PWLLs and through-knee PWLLs. It is interesting to observe that the distribution of TF and TK between the legs which could find a solution in the reduced endurance state (TF = 3, TF = 4) and the legs which could not find a solution (TF = 3, TK = 2) was fairly even. This is unexpected, as the TF legs will have a lower residual muscular capacity, suggesting that the properties of the more proximal flexor muscles (such as the iliacus and psoas major) are responsible for this distinction between the legs which could maintain kinematics in the reduced endurance state and the legs which could not.

There are limitations to this study, notably that the inverse dynamics model is limited to the recorded kinematics. This means that any kinematic changes that may occur due to the simulated reduction in force capacity in the *reduced endurance* condition were not included. A forward dynamics model could predict the changes in kinematics due to the potentially reduced endurance of certain muscles. A forward dynamics model could also predict optimal kinematics for reduced muscle activation and improved load sharing. Predicted optimal kinematics could be employed as a quantified aim in the design of a novel rehabilitation intervention. Forward dynamics could also be used to identify the cause of the variability in muscle activations observed in this study by modeling differences in anatomy (due to surgery, training, or natural variation) and biomechanics. Other limitations include the inherent assumptions and estimates of the MSK model. For example, although the muscle volumes were subject-specific, the fiber length to muscle length ratio, pennation angle, and sarcomere length were obtained from *in vivo* cadaveric studies (Toderita et al., 2021) as it is not possible to acquire these values from MRI imaging. Muscle force capacities are sensitive to such properties. $F_{\max}^{i}$ has been used throughout this study as an indication of force capacity; these values were also adapted to account for atrophy and hypertrophy, as previously described, in a subjective manner. Although this study did not include the dynamic properties of muscle activation and contraction with respect to length and velocity, this limitation is mitigated by the slow nature of the task studied here. The literature shows that inclusion of these properties results in similar magnitudes of muscle force for gait when compared to our static optimization approach (Anderson and Pandy, 2001; De Groote et al., 2016).

This study has developed a new approach to analyze muscular endurance using inverse dynamics modeling. It has highlighted the critical muscles that are likely to have reduced endurance and so limit PWLLs’ ability to walk. Research on clinical interventions to address this reduced endurance may focus on gait retraining, such as changing the kinematics or modifying the balance of muscle forces required to achieve a proscribed gait pattern.

## Data Availability

All data are available upon reasonable request, subject to the ethical approval and participant confidentiality. Requests to access these datasets should be directed to a.benton20@imperial.ac.uk.
